# Mixed-Up-Ness or Entropy?

**DOI:** 10.3390/e24081090

**Published:** 2022-08-08

**Authors:** W. Seitz, A. D. Kirwan

**Affiliations:** 1 Department of Marine Sciences, Texas A&M University at Galveston, Galveston, TX 77553, USA; 2 School of Marine Science and Policy, University of Delaware, Newark, DE 19716, USA

**Keywords:** entropy, majorization, incomparability, Young Diagram Lattice, mixed-up-ness, 01.55.+b, 01.70.+b, 05.50.+q, 05.70.−a

## Abstract

**Simple Summary:**

The second law of thermodynamics has a mystical appeal in disciplines with tenuous connections to its origins. We hypothesize that many of these appeals instead should be to another principle heretofore unrecognized: the law of mixed-up-ness (LOM). Instead of using a number such as entropy to characterize randomness, non-thermodynamic systems can be arranged in simple diagrams according to their degree of mixed-up-ness. Curiously, the evolution of such systems from degrees of low to high mixed-up-ness is both consistent with and richer than the principle of increasing entropy.

**Abstract:**

*Mixed-up-ness* can be traced to unpublished notes by Josiah Gibbs. Subsequently, the concept was developed independently, and under somewhat different names, by other investigators. The central idea of mixed-up-ness is that systems states can be organized in a hierarchy by their degree of mixed-up-ness. In its purest form, the organizing principle is independent of thermodynamic and statistical mechanics principles, nor does it imply irreversibility. Yet, Gibbs and subsequent investigators kept entropy as the essential concept in determining system evolution, thus retaining the notion that systems evolve from states of perfect “order” to states of total “disorder”. Nevertheless, increasing mixed-up-ness is consistent with increasing entropy; however, there is no unique one-to-one connection between the two. We illustrate the notion of mixed-up-ness with an application to the permutation function of integer partitions and then formalize the notion of mixed-up-ness as a fundamental hierarchal principle, the law of mixed-up-ness (LOM), for non-thermodynamic systems.

## 1. Introduction

The conservation of mass/energy, charge, and momentum are universally regarded as fundamental laws of physics and thus cannot be deduced from a more basic set of principles. They are equalities that apply to discrete corporeal objects. The equation statements of these laws are indifferent to the direction of time, and they specify state variables such as mass, charge, and velocity, which can be measured with high degrees of precision.

A common statement of the second law of thermodynamics (SL), is that for every system in equilibrium the entropy change of the system and its surroundings, considered together, is positive and approaches zero for any process that approaches reversibility. Beginning with tedious experiments on heat-engine efficiencies at the start of the industrial revolution, this law rose to become the superior of all scientific laws. Sir Arthur Eddington succinctly stated this point of view in his famous quote:

*The law that entropy always increases holds, I think, the supreme position among the laws of nature. If someone points out to you that your pet theory of the universe is in disagreement with Maxwell’s equations—then so much the worse for Maxwell’s equations. If it is found to be contradicted by observations—well, these experimentalists do bungle things sometimes. But if your theory is found to be against the second law of thermodynamics, I can give you no hope; there is nothing for it but to collapse in deepest humiliation.*

The second law differs in two quintessential ways from the other fundamental laws. First and foremost, it is an inequality. References [1,2], and the incisive review of [3] provide excellent synopses of the scientific and philosophical issues regarding the SL inequality and the arrow of time. Second, in contrast to the state variables of other fundamental laws, classical thermodynamic theory is ambiguous about how to measure entropy. Grad [4] perceptively noted that there are many entropies. In both classical thermodynamics and statistical mechanics, entropy change only determines precedence of states, but is silent about when they will occur. See also Klika et al. [5,6] for more recent discussions on multiscale interpretations of entropy.

There are two other subtle but significant differences between the SL and all other fundamental laws. First, there is the relationship between the SL and statistical mechanics, which differs from the relationship between classical and quantum mechanics. In the latter case, the correspondence principle relates the expected values of momentum and position obtained from Schrödinger’s equation with Newton’s laws. This establishes a consistency relation between quantum mechanics and Newton’s laws, not a proof that Newton’s laws are based on quantum mechanical principles. This stands in contrast to a common belief that the SL is based on statistical mechanical principles.

The other distinction, our prime motivator here, is the breadth of disciplines that claim some ontological connection to the SL yet provide no link to energy or the first law of thermodynamics. Ben-Naim [7] has provided an incisive review and commentary of many examples.

In reviewing some of these examples, several overarching themes emerge that have no obvious connection to thermodynamics. First, they often refer to ethereal or conceptual entities. In contrast, thermodynamics and statistical physics deal with corporeal entities that are readily measured. They attempt to characterize the tendency of many systems to evolve towards randomness; hence, the appeal to the statistical mechanical concept of entropy. Applicants have a need to characterize an order or sequence in inordinate data sets that are independent of thermodynamics, statistical mechanics, or irreversibility. Establishing order requires an inequality, but irreversibility generally is irrelevant in these applications. Finally, contrary to the origins and use of the SL where there are close connections with energy flow, energy considerations in the appeals are usually indirect, after the fact, or even absent.

The disconnect between references to the SL independent of classical thermodynamics or statistical mechanics was recognized by Denbigh [8], who laconically noted, “There are many entropies and not all of them are related to the second law of thermodynamics”. One also is reminded of the wry suggestion by John von Neumann to Claude Shannon (see [9]) on what to name the latter’s information measure:

*You should call it entropy, for two reasons. In the first place your uncertainty function has been used in statistical mechanics under that name, so it already has a name. In the second place, and more importantly, no one really knows what entropy really is, so in a debate you will always have the advantage.*

Von Neumann recognized that Shannon’s information function was proportional to that used in statistical physics since the time of Boltzmann. While it is reasonable to broaden the meaning and use of scientific terms—consider the vernacular uses of “energy” and “momentum”—we question whether entropy is the appropriate descriptor of the evolution of non-thermodynamic systems. In traditional thermodynamic applications of the SL there are other parameters, such as temperature, pressure, etc., that impact the evolution of these systems. This is not the case for non-thermodynamic SL appeals since they rarely, if ever, specify additional factors that could govern system evolution.

Our proposal is to use mixed-up-ness instead of entropy as the critical diagnostic to gauge the evolution of non-thermodynamic systems. Mixed-up-ness differs fundamentally from entropy. Entropy is a number whose value depends on user-specific algorithms; mixed-up-ness is depicted as a diagram, specifically a Young Diagram as discussed in detail by Ruch and Mead [10]. There is no ambiguity in Young Diagrams. The increase in mixed-up-ness is given by a Young Diagram Lattice (YDL), a sequence of Young Diagrams determined by majorization.

Does this connect with classical thermodynamics and statistical mechanics, which emphasizes entropy as the diagnostic variable? Jaynes [11] established a rigorous connection between Shannon’s formulation and Gibbsian statistical mechanics. In reviewing Jaynes’ work, Dias and Shimony [12] stated that the maximum entropy principle advocated by Jaynes

*... is uniquely determined as the one which is maximally noncommittal with regard to missing information and it provides the most honest description of what we know. Between this quite plausible justification and the residual suspicion that somehow too much knowledge has been extracted from ignorance there may appear to be a deadlock.*

It is our view that extracting too much knowledge from ignorance is a serious yet unrecognized issue in non-thermodynamic references to the SL. In a vast majority of these references, a Gibbsian statistical mechanics version of entropy characterizes randomness or disorder. We have two concerns. First, as preceptively suggested by [12], entropy may not be a natural gauge of ignorance. Second, the Boltzmann entropy, the most commonly used version of entropy in non-thermodynamic applications of the SL, has numerous degenerate or isentropic states [13]. In traditional thermodynamics, there are state variables such as temperature and pressure, etc,. that govern system evolution. In these cases, degenerate entropies may not be a serious impediment in diagnosing system evolution. This is not the case for non-thermodynamic SL applications since they generally do not specify other factors that could govern system organization/evolution.

So how can ignorance be quantified, and what is a suitable state variable for our approach? We suggest majorization is the key, as it establishes all states that are accessible via the YDL from any prescribed state. However, often there are many states that cannot be accessed from the YDL by a given state. Simply put, a given state is ignorant of such states. Hence, the number of inaccessible states of a given state is an appropriate state variable.

Our hypothesis is that appeals to the SL with tenuous connections to thermodynamics or statistical mechanics are misdirected. Instead, they should be to a different principle with no connection to energy, thermodynamics, or statistical mechanics. The primary goal in this communication is to put forward a precise statement of such a principle, yet is still consistent with the notion of ordering by entropy. To this end, the balance of our report is organized as follows. Section 2 reviews three disparate lines of research that point to such a principle. Section 3 introduces the notion of mixed-up-ness with a simple example and reviews the essential mathematical details of integer partitions (IP), hereafter referred to as IPs. IPs are a logical choice to illustrate the LOM because they are readily connected to the Boltzmann entropy, which has wide applications in science and engineering. See, for example, Refs. [14,15,16,17] and references cited therein. Section 4 explores the connection between mixed-up-ness and IPs. Section 5 provides an example of how mixed-up-ness can organize a large relational database. Section 6 summarizes our analysis and discusses metaphysical implications of the LOM.

## 2. Background

Perhaps the first to recognize the need for an entropy principle without recourse to classical thermodynamics was Josiah Gibbs [18]. Although Gibbs’ notes on this were unpublished, his notion of mixed-up-ness seems to us to be the appropriate descriptor and is adopted here.

Later, Ernst Ruch and associates [10,19,20,21,22] developed and applied a mixing schema based on the majorization partial order and the Young Diagram Lattice (hereafter YDL). This approach was demonstrated to be consistent with a monotonic increase in entropy as prescribed by the second law of thermodynamics; however, it does not involve energy. In Section 3, we return to Ruch’s analysis of mixing, here termed mixed-up-ness.

Finally, in 1999, Lieb and Yngvason [23] argued that since the SL is recognized as a fundamental principle it does not require any statistical or quantum foundation. Subsequent publications [24,25,26] developed a conceptual basis for the SL without reference to either statistical mechanics or heat engines. Hereafter, we often refer to this body of work as LY.

A critical aspect of LY is the comparison of different equilibrium states via “adiabatic accessibility” to determine the order, or precedence, in which they occur. In Ref. [24], they assert that an equilibrium state *X* precedes an equilibrium state *Y* if and only if an entropy function S(X)≤S(Y) exists. For our purposes, it is sufficient to simply require that *X* precedes, or can evolve to, *Y*. We stress that the precedence schema of LY does not specify time, much less a direction of time. Their cynosure is the order of occurrence, or precedence. Note that LY, [19], Gibbs [18], and our development here are mute concerning reversibility/irreversibility. As shown by Pavelka et al. [27] in the General Equation for Non-Equilibrium Reversible-Irreversible Coupling (GENERIC) formulation of thermodynamics entropy alone is not sufficient to gauge reversibily/irreversibility.

## 3. Mixed-Up-Ness and the Mathematics of Integer Partitions

The notion of mixed-up-ness and IPs has a curious history. It began with the study of partitions of integers, where a partition of a positive integer *N* is a multiset of positive integers that sum to *N*. The first important theorem regarding partitions is due to Euler [28], the father of partition theory. One hundred and fifty years later, R. F. Muirhead [29] developed a partial order among partitions known as majorization. At approximately the same time, Alfred Young demonstrated that a diagrammatic representation of majorization is a mathematical lattice, now often referred to as the Young Diagram Lattice (YDL).

While studying chirality, Ruch [19] introduced a general concept, which he called mixing, that applies to any set of objects that can be divided into subsets without objects in common. The partitions of integers are all possible subsets of integers that sum to *N*. Using a theorem from Hardy et al. [30], Ruch proved that mixing and the majorization partial order are mathematically equivalent.

The concept of the mixed-up-ness partial order is illustrated by a simple example. Consider 4 baskets of fruits, each containing different combinations of apples, pears, and oranges. Suppose further that 2 baskets contain 4 apples and 2 pears each, and the other 2 baskets have 4 apples and 2 oranges each. Now, mix the four baskets together in one large basket. This gives a basket containing 16 apples, 4 pears, and 4 oranges. This is equivalent to four baskets each containing 4 apples, 1 pear, and 1 orange. As this was obtained by mixing the original 4 baskets, we conclude that 4 apples, 1 pear, and 1 orange is more mixed up than the original baskets. Or, a 4, 1,1 combination is more mixed than a 4, 2 combination. However, if we repeat this procedure with a 3, 3 combination of fruits for the 4 baskets, it is straightforward to see that it is impossible to obtain a 4, 1, 1 basket of anything from any 3, 3 combination, and vice versa. The significance that some combinations are neither more nor less mixed up is shown later.

**Figure 1:**
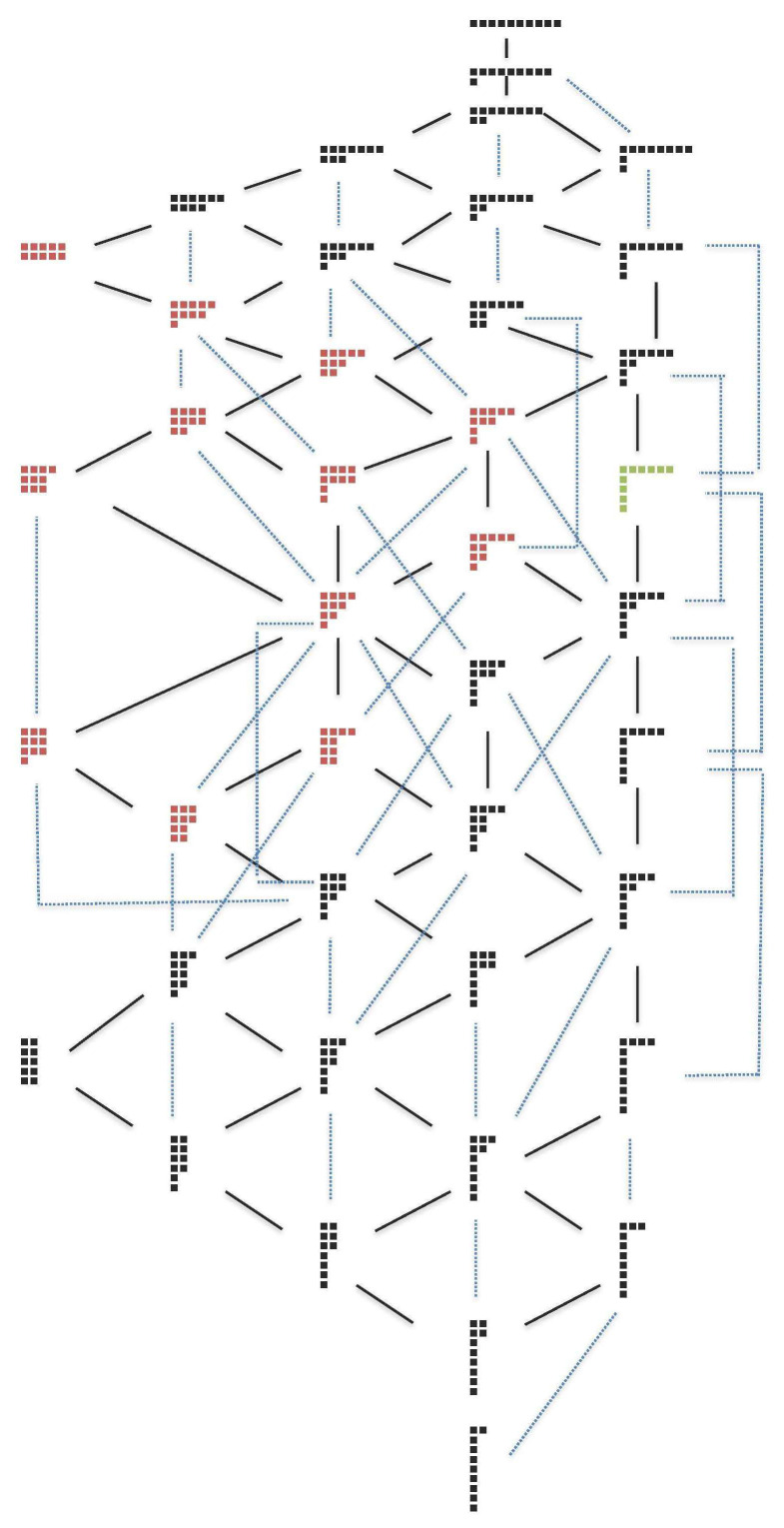
This figure shows the lattice for 41 of the 42 Young Diagrams. The last Young Diagram is not shown. See text for further explanation.

To make the idea of mixed-up-ness more precise, we write the *j*th partition of *N* as $p_{j}=\left[n_{1j},n_{2j},\cdots ,n_{Nj}\right]$ with $\sum_{i=1}^{N}n_{ij}=N$ for every *j*, $n_{ij}\geq n_{i+1j}$, and $j=1,\cdots ,N_{*}$, where $N_{*}$ is the total number of partitions of *N*. For *N* = 10, $N_{*}$ = 42 (see Figure 1, for the associated YDL). Hereafter, we simplify the notation so that partition elements that are 0 are not demonstrated and repeated elements are indicated by an exponent that indicates the number of repetitions. For example, for N=10 the partition [3,2,2,1,1,1,0,0,0,0] is written as $\left[3,2^{2},1^{3}\right]$. We characterize any partition $p_{j}$ by 3 integers. The first is obvious: The sum of all partition elements is *N*. The second is the partition permutation number given by

(1) $$\Omega_{j}=\frac{N!}{\prod_{i}^{N}n_{ij}!}\;\;\forall \;\;j=1,\cdots ,N_{*}.$$

This is recognized as the number of ways the *N* elements of $p_{j}$ can be arranged in the partition. $\Omega_{j}$ also has a foundational role in statistical physics since its logarithm is proportional to the Boltzmann entropy. The third is incomparability, discussed below.

A curious and important feature of IPs is that for N≥7 there are some $\Omega_{j}$ that are the same, i.e., they are degenerate. N=7 provides a simple example. Here, the partitions $\left[4,1^{3}\right]$ and $\left[3,2^{2}\right]$ have the same permutation number, 210. Moreover, for large *N* most $\Omega_{j}$ are degenerate [13]. This turns out to be a crucial aspect of our analysis that was not recognized by Gibbs [18]), Ruch [19], or Lieb and Yngvason [23]. The degeneracies imply partial ordering for those entropy values, which leads naturally to majorization and incomparability.

Any vector can be partially ordered by majorization. Moreover, the partitions in the permutation function, (1) are IPs, hence we need only to consider the special case where the vectors are IPs. To define the majorization of IPs, consider two partitions $p_{j}=\left[n_{1j},n_{2j},\cdots ,n_{Nj}\right]$ and $q_{j}=\left[m_{1j},m_{2j},\cdots ,m_{Nj}\right]$. Partition $p_{j}$ is said to majorize partition $q_{j}$, symbolized as $p_{j}≻q_{j}$, if

(2) $$\sum_{i=1}^{k}n_{ij}\geq \sum_{i=1}^{k}m_{ij}\;\;\forall \;\;k=1,\cdots N;\;\;j=1,\cdots N_{*}.$$

If $p_{j}$ does not majorize $q_{j}$ and $q_{j}$ does not majorize $p_{j}$, these two partitions are termed *incomparable*. As an example, it is readily observed that the two partitions $\left[4,1^{2}\right]$ and $\left[3^{2}\right]$ in the fruit basket example discussed above are incomparable. The physical implication is that if $p_{j}$ and $q_{j}$ are incomparable, then $p_{j}$ cannot evolve to $q_{j}$ and vice versa.

From (1) and (2) it follows that if

(3) $$p_{j}≻q_{j}\;\mathrm{then}\;\Omega \left(p_{j}\right)<\Omega \left(q_{j}\right).$$

In the [24] schema, this result is not a trivial matter. We return to this issue in Section 5.

Partial ordering leads to the third property considered here, the incomparability number $C_{j}$. This is the number of partitions that are incomparable to a partition $p_{j}$. $C_{j}$ can be determined arithmetically by merely counting the number of partitions that do not satisfy (2) for partition $p_{j}$. Alternatively, there is a geometric approach, which proves to be more appropriate for directed lattices. In the graph theory of partially ordered sets, the YDL (see Figure 1) is a directed graph or digraph, which is a lattice. *Edges* in a directed graph connect neighboring vertices or nodes. A walk on a graph is a finite or infinite sequence of nodes. A trail is a walk in which edges are distinct, and a path is a trail in which all vertices (consequently all edges) are distinct [31]. For the YDL, two nodes, i.e., partitions in the present case, are comparable if there is a path between them and incomparable if not. Consequently, paths along the YDL only visit comparable partitions. In Figure 1, paths follow solid black lines.

These ideas are illustrated in Figure 1, which shows the YDL for N=10. The green colored Young Diagram in this figure ($\left[6,1^{4}\right]$) has the highest incomparability number $C_{j}$ of 12. The incomparable partitions are shown in red. The permitted transitions under majorization in this figure are shown by black lines, while the blue lines are not permitted [32]. By inspection, one finds that the shortest paths for N=10 on the YDL visit 16 partitions while the longest visit 20 partitions.

What is the connection between the $C_{j}$ and the comparison of equilibrium states by adiabatic accessibility discussed by [24]? In a physicist’s view, every partition is an equilibrium or observable state, and the counterpart of adiabatic accessibility is comparability by majorization. That is, if $p_{j}≻q_{j}$, we claim that $p_{j}$ precedes, or can evolve to $q_{j}$ through majorization. Clearly, incomparable partitions do not meet this criterion. So even though $\left[4,1^{3}\right]$ and $\left[3,2^{2}\right]$ have the same permutation number, they are not accessible to each other. Put another way, if $p_{j}$ and $q_{j}$ are incomparable then they are ignorant of the existence of each other. As previously observed, many $p_{j}$ may have the same $\Omega_{j}$, and we have found that a few of these partitions may also have the same $C_{j}$.

The information on partitions quickly accumulates simply because of the rapid increase in the number of partitions with *N*. To categorize this large amount of information, we appeal to the notion of relational databases as developed by [33]. Relational databases arrange information into tables. Each row of a table contains a record or a tuple and a unique key. Rows in a table can be linked to rows in other tables by adding a column for the unique key of the linked row. Data relationships of arbitrary complexity are now routinely treated by these concepts.

For IPs of any *N*, there is a relational database $D_{0}$ composed of 3 tables. These are P, a list of every partition $p_{j}$; Ω, the permutation numbers $\Omega_{j}$ for all $N_{*}$ partitions; and C, the incomparability numbers $C_{j}$ for those partitions. Each table has $N_{*}$ records, all entries in the tables are integers, and the order of the records retains the 1:1 relationship between P, Ω, and C. That is,

(4) P≎Ω≎C.

We return to the IP database in Section 5.

## 4. The Law of Mixed-Up-Ness for Integer Partitions

As stated in the introduction, IPs are especially appropriate for an application of the LOM. In this section, we explore the philosophical connections between IPs and the notion of mixed-up-ness. It is convenient to take two complimentary points of view. One is a static approach that focuses on characterizations of mixing states, effectively formalizing the discussion on mixing in Section 3. The other is a dynamic approach, which relies on the YDL to establish sequences of the partitions. Semantically, these two approaches are based on the use of *mix* as a noun or verb.

**Figure 2:**
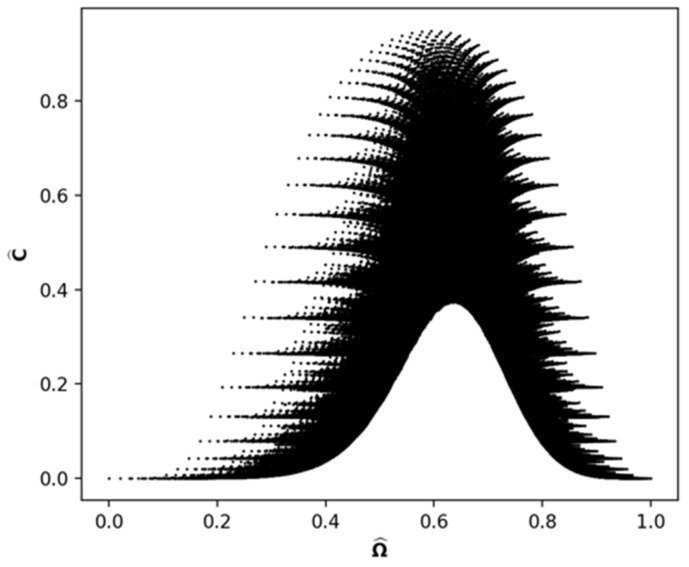
Mixing space for N=61, the scatter plot of $\hat{C}$ (Scaled Incomparability Number) vs. $\hat{\Omega }$ (Scaled Permutation Number).

As a noun, *mix* specifies the number of each type of objects in a mix. This is represented as a single partition. The relational database $D_{0}$ contains the information for all possible mixes of *N* objects. In this database, P is the table of partitions; Ω is the table of permutation numbers for each partition obtained from (1); and C is the table of incomparability numbers for each partition obtained from exhaustive calculations using (2). For N=61 there are 1,121,505 entries in each of these tables. This information is visualized as a plot of $\left(\Omega_{j},C_{j}\right)$ in Figure 2, which we call the *mixing space* for N=61. Because of the huge range in values, the abscissa is scaled as $\hat{\Omega }={\left(\ln N!\right)}^{-1}\ln\Omega$ and the ordinate as $\hat{C}=N_{*}^{-1}C$. This is the static view of a mix.

Two features of Figure 2 are particularly noteworthy. The first is the huge range of incomparable partitions. All partitions, other than the first and last three along the abscissa, have nonzero incomparability numbers. Moreover, the range of the incomparability numbers can be quite large, especially for those near the midpoint of the abscissa. The other noteworthy feature is the bell shape of the upper and lower boundaries and the density of partitions between these boundaries in the midrange of $\hat{\Omega }$. The density of partitions shows there are many values of $\hat{C}$ for any given value of $\hat{\Omega }$ and vice versa. The large range of $\hat{C}$ for a given $\hat{\Omega }$ is of particular interest.

**Figure 3:**
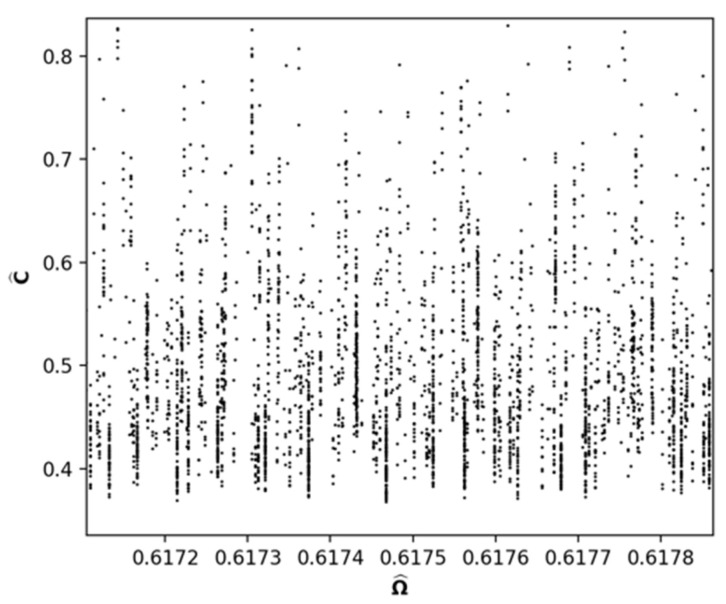
Slice of Figure 2 for the region $0.6172<\hat{\Omega }<0.6178$. The vertical stripes are degenerate $\hat{\Omega }$.

Figure 3 shows an extremely thin slice of Figure 2 between $0.6172<\hat{\Omega }<0.6178$. The noteworthy feature of this figure is the vertical stripes indicating the presence of degenerate $\hat{\Omega }$ or doppelgängers [13]. This suggests that the thickness of Figure 2 is due to a large number of degenerate permutation numbers.

Data for N=61 illustrate the significant role of degenerate entropies. The total number of partitions is given by

(5) $$N_{*}=\sum_{d=1}^{dmax}n_{d}.$$

where *d* is the degeneracy, $n_{d}$ is the number of partitions of degeneracy *d*, and dmax is the maximum degeneracy. For N=61 $N_{*}$ = 1,121,505, dmax=206, $n_{1}=31,054$, $n_{2}=$ 31,242, $n_{3}$ = 36,045, $\cdots ,n_{dmax}$ = 206. The number of distinct permutation numbers is given by

(6) $$N_{u}=\sum_{d=1}^{dmax}n_{d}/d.$$

For N=61 $N_{u}$ = 119,103.

As a verb, *mix* describes a dynamic process that changes the makeup of a system, in our case, a process that converts one partition to another. To study this, we refer to the YDL (see Figure 1) for the case of N=10. In the YDL, each node is a partition, and the YDL shows the allowed transitions from one partition to the next in the partial order. For much of what follows, we consider paths that travel from partition [N] to partition $\left[1^{N}\right]$; recall the definition of a path given in Section 3. It is clear from (3) that the $\Omega_{j}$ increase monotonically along every path. However, the $C_{j}$ do not. Below we argue that while entropic considerations lead to the SL, the inclusion of both incomparability number $C_{j}$ and permutation number $\Omega_{j}$ lead to another principle, the LOM.

In order to connect our IP application to LY, we regard each partition as an equilibrium state. Thus the question: How might the partitions be organized to provide some sense of coherence?

To do this we appeal to the “single-shot statistical mechanics” approach [34,35]. One aspect of this approach questions von Neumann entropy in non-equilibrium systems. Egloff et al. [35] state, “We argue it (majorization) should therefore be the central quantity of statistical mechanics, rather than the von Neumann entropy”. This was recognized much earlier by [19] in his statement of the principle of increasing mixing character:

*The time development of statistical (Gibbs) ensemble of isolated systems (microcanonical ensemble) proceeds in such a way that the mixing character increases monotonically. Increase of mixing character is equivalent to increase of mixing disorder and decrease of statistical order.*

Ruch’s principle of increasing mixing character neither requires energetics nor statistical mechanics. Both Ruch and Egloff conclude that majorization determines precedence.

Seitz and Kirwan [36] studied paths on the YDL from partition [N] to partition $\left[1^{N}\right]$ and found from standard lattice dynamic Monte Carlo simulations [37] that the distribution of path lengths *L* scaled as $L∼N^{4/3}$. Thus, an individual YDL experiment samples a small fraction of the relational database $D_{0}$.

**Figure 4:**
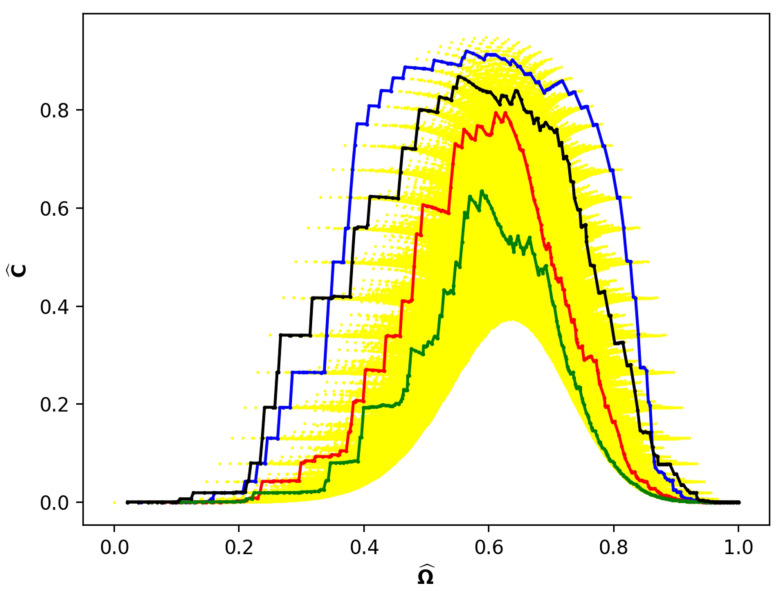
Four example paths in the mixing space of N=61 superposed on Figure 2. Blue path is length 197, black path is length 206, red path is length 254, green path is length 294.

Figure 4 shows four example paths, corresponding to four single shot experiments, superimposed on Figure 2. The path lengths vary from 197 to 294. Here, we show paths in the mixing space ($\hat{\Omega_{j}}$, $\hat{C_{j}}$) since paths on the YDL itself cannot be demonstrated because the number of nodes is greater than $10^{6}$.

Shorter paths occupy states along the outer portions of Figure 2, whereas the longer paths visit the inner regions. Two aspects of this figure seem noteworthy. The first is the wide variation in incomparability both along each path and between the paths. Apparently, there is little correlation between the maximum incomparability of individual paths and the maximum incomparability of the mixing space. The second is the intersection of the paths at both low and high values of $\hat{\Omega }$. However, at intermediate values there appears to be a general separation of the paths.

**Figure 5:**
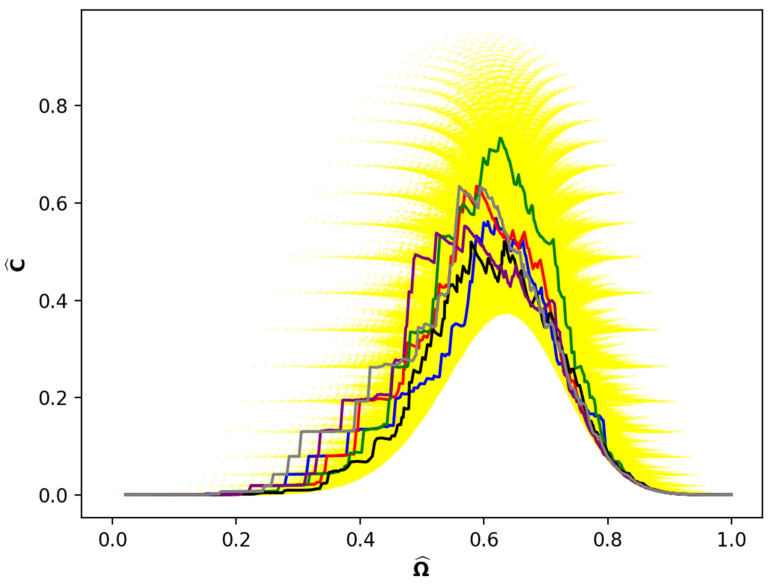
Six example paths in the mixing space of N=61 of length 294 superposed on Figure 2.

Figure 4 illustrates a diversity of paths of different lengths. What about paths that have the same length? Figure 5 shows six representative paths all of length 294. The size of the area of the scatter plot covered by this small number of sample paths and the consequent variability in comparability is surprisingly large, although neither are so great as in Figure 4. Perhaps the most remarkable characteristic of Figure 5 is that the paths intersect at many values of $\hat{\Omega }$. The paths cross each other numerous times in this small interval as well as are parallel or completely overlap for brief segments of $\hat{\Omega }$. We call this a tangle of paths.

**Figure 6:**
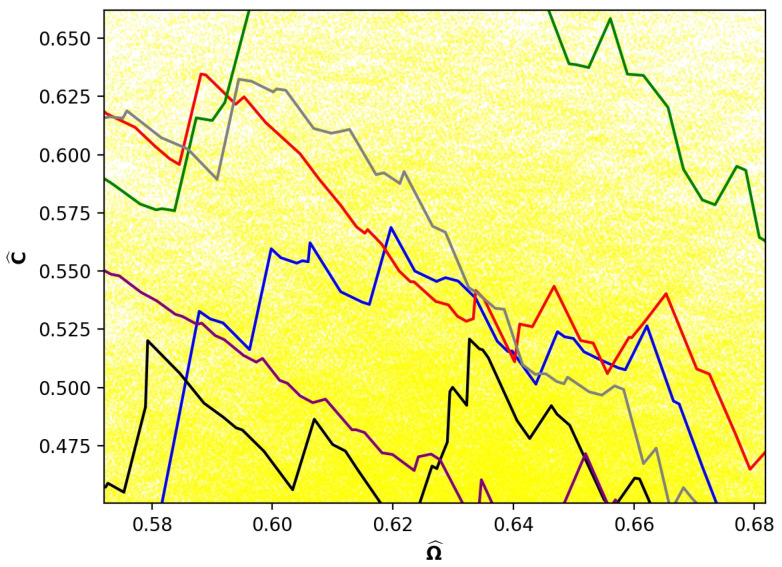
Slice of 6 paths shown in Figure 5 in region $0.58\leq \hat{\Omega }\leq 0.68$.

The tangles, along with a variation in incomparability, are illustrated in more detail in Figure 6, which is a slice near the region of maximum incomparability in Figure 5. Note, in particular, the large number of intersections of the different paths in a narrow region of $\hat{\Omega }$.

Figure 4 and Figure 5 are a compilation of just 10 Monte Carlo-simulated paths. Although the paths vary in length and cover a swath of the mixing space shown in Figure 2, it is clear that many more simulations would be needed to sample all partitions.

## 5. Analysis of the Integer Partition Relational Database

We return now to the database discussed in Section 3. The table of partitions P, of $D_{0}$, is ordered from 1 to $N_{*}$ according to whatever algorithm was used to generate the partitions. Tables Ω and C are obtained by the permutation number and incomparability number for each partition in P, but they are not in any particular order. However, as stated by (4), they retain a 1:1 relationship with their respective partitions.

What is the connection within the database $D_{0}$ (composed of 3 tables of apparently randomly sorted records) that is relevant to the LY development of a second law of thermodynamics independent of heat engines and statistical mechanics? To answer this question, we appeal to three demons charged with establishing some sense of structure to $D_{0}$.

An “entropy” demon links P to Ω by an unique key and sorts Ω in non-decreasing order while retaining the links to P. In the sorted Ω clearly

(7) $$\Omega_{j+1}-\Omega_{j}\equiv ▵\Omega_{j}\geq 0,\;\;j=1,\cdots ,N_{*}-1.$$

This provides some semblance of order to the original database $D_{0}$. ***It*** calls this new database $D_{1}$. However, as noted earlier, many $\Omega_{j}$ are degenerate and hence there are many partitions linked to a single value of $\Omega_{j}$. This prompts the demon to postulate the existence of “isentropic” processes to account for degenerate $\Omega_{j}$.

A second “lazy” demon does not accept the idea of isentropic processes. In the spirit of LY, ***It*** opts to rely on “real world experience” and elects to perform an ensemble of “single-shot” experiments. As Ω increases, when each degenerate $\Omega_{j}$ occurs, the experiment selects only one. ***It*** then concludes

(8) $$▵{\tilde{\Omega }}_{k}>0,\;\;k=1,\cdots ,N_{u}-1$$

for every ensemble member *k*. Here, $N_{u}$ is the number of distinct values of $\Omega_{j}$ given by (6). The lazy demon produces an ensemble of databases $D_{2}$, each of which has two tables: one $\tilde{\Omega }$ containing the unique values of $\Omega_{k}$, the other $\tilde{P}$ where the ${\tilde{P}}_{k}$ are determined by single-shot experiments for each degenerate $\Omega_{j}$.

As noted previously for N=61, $N_{u}$ = 119,103 or about 10% of the total number of partitions, but the strict inequality of (8) precludes isentropic processes implied by the first demon and thus is in fundamental disagreement with (7).

Although the entropy demon’s conclusion is consistent with the LY stipulation that ▵Ω≥0, it apparently is not necessarily consistent with their notion of precedence determined by adiabatic accessibility. There are many partitions with the same $\Omega_{j}$, which neither LY nor [19] considered. The lazy demon’s analysis attempted to address this issue, but because of the necessity to examine all ensemble members of $D_{2}$ ***It*** incurred enormous additional computational effort.

An enlightened third demon notes that neither of the previous two demons utilized any information from table C. This demon observes that while entries in Ω increase monotonically in $D_{1}$, the entries in C peak near the midpoint of $\hat{\Omega }$; see Figure 2. Furthermore, ***It*** recalls that table C was constructed from the majorization partial order, which provided incomparability numbers for each partition. ***It*** has an epiphany and realizes that in all cases precedence is determined by majorization rather than by entropy. ***It*** then develops new databases $D_{3}\left(k\right)$, where *k* ranges from 1 to the number of paths in the YDL. For each path, the records of Ω are sorted by majorization. Upon reading the files, ***It*** recognizes that in each of these databases

(9) $$▵{\vec{\Omega }}_{l}>0,\;\;l=1,\cdots ,L.$$

Here, *L* is the path length. The paths in Figure 4 and Figure 5 are examples of what may be recorded while ***It*** reads the records.

With some astonishment, the enlightened ***It*** notes that (9) agrees with (8). Moreover, any single shot experiment incurs less computational cost than (8) since $L∼N^{4/3}$. Most importantly, by using the information in table C to select paths based on the majorization partial order instead of selecting partitions based on increasing entropy, ***It*** resolves the discrepancy between (7) and (8) by noting the existence of numerous degenerate $\Omega_{j}$. Furthermore, (9) demonstrates that the majorization criterion is consistent with the notion of adiabatic accessibility and the contention in [19] that it is also consistent with the second law of thermodynamics.

Three lessons are drawn from this parable:

1. The precedence of states is the critical aspect of both the SL and the LOM.
2. Majorization is consistent with the notion of adiabatic accessibility and establishes precedence of partitions.
3. Energy need not be involved in establishing precedence.

## 6. Discussion

Note that (9) resembles the second law of thermodynamics for isolated systems. However, it arises from a fundamentally different and simpler approach than that used in statistical mechanics. Recall in the latter, microstates are prescribed in a six dimensional phase space with particles located in cells characterized by discrete energy levels. Contrast this with the LOM, where microstates are prescribed by $\Omega_{j}$, the anti-log of the Boltzmann entropy, which is characterized by the IPs of the number of objects in a system. There is no need to specify energy. Moreover; the schema is exact, can be applied to any *N*, involves only integers, and precedence is established by majorization.

The classic thermodynamic gauge for assessing the evolution of systems is increasing entropy. This was the approach used by the entropy and lazy demons. However, the approach used to obtain (9) is fundamentally different. Here, increasing mixed-up-ness, rather than entropy increase, is the criterion for assessing evolution priority. As demonstrated by the lazy demon, the entropy gauge alone identifies ambiguous paths. In contrast, the mixed-up-ness gauge produces numerous specific paths. Yet every one of these paths is consistent with the SL. Thus there are two essential distinctions between the SL and the LOM. First, there are no energetic requirements for system evolution, and second, precedence is determined by increasing mixed-up-ness.

Our development parallels the approach taken by LY, in the sense that we also make no reference to energy or heat reservoirs, nor do we make use of statistical mechanics. The approach in [23] is based on a concept of precedence. They argued that the equilibrium state *X* precedes the equilibrium state *Y* if and only if there is an entropy function whose value at *X* is less than or equal to its value at *Y*. They [24] also observed that the converse assertion is “not guaranteed *a priori*..., but is empirically testable and appears to be true in the real world”. Note that their analysis assumes that either *X* precedes *Y* or *Y* precedes *X*. If equilibrium states *X* and *Y* are partially ordered, however, there is a third possibility, namely than neither is greater than the other, thus they are incomparable. Mixed-up-ness of partitions is such a partial order and majorization is the criterion for determining precedence among them.

The LOM differs in two key respects from the approach taken by LY. First, from (3), it is clear that the converse statement about precedence and entropy is trivially true and does not require any real world experience. Second, as demonstrated in Figure 3, there are many degenerate $\Omega_{j}$, a possibility not considered by either LY or [19]. This possibility was anticipated by [38], who postulated that for any equilibrium state there are nearby equilibrium states that are adiabatically inaccessible. Clearly, entropy is not a unique characterization of precedence.

As noted previously, [35] concluded that majorization rather than von Neumann entropy is the central quantity in statistical mechanics. While their conclusion is consistent with our use of majorization to establish precedence, their focus was on one-shot experiments, which relied on statistical mechanics and energetics. A single path through the IPs performed by the third demon is analogous to a single-shot experiment. A large number of paths would recover a figure quite similar to Figure 2.

In contrast to LY, [19] proposed a principle for the evolution of systems based on the concept of increasing mixing character. He argued convincingly that mixing is a “quality” rather than a “quantity”. His principle guarantees precedence without resorting to real world experience. Ruch left open the question of the existence of a “set of mixing homomorphic functions sufficient to specify increasing mixing character” but without resorting to a partial order. No such functions have yet been found. He [19] also proved that the criterion to determine whether or not a partition is more or less mixed than another is majorization. This demonstrates that majorization is a fundamental physical principle. As shown by the third demon, accessibility via majorization differs fundamentally from accessibility based on entropic order, yet it always selects precedence consistent with an increase in entropy.

Are there metaphysical implications of the LOM vis-à-vis the SL? The latter deals with systems composed of corporeal objects and connects the evolution of macroscopic states of these systems to energy. There is a presumption among thermodynamicists that precedence is ultimately based on entropy (however it may be defined). It seems to us that the LOM is a more basic principle for two reasons. It can be applied to statistical physics as demonstrated by [35] without contradicting the SL. In addition, it is applicable to disciplines that have no direct connection to statistical mechanics and energetics.

What has the analysis of the LOM to IPs taught us? First, precedence is determined by mixed-up-ness, rather than by entropy. Nevertheless, mixed-up-ness precedence is consistent with the second law requirement of increasing entropy. Second, in our example, increasing mixed-up-ness was determined by majorization. Majorization establishes a new “ignorance” function: the number of incomparable states/partitions that are inaccessible along a prescribed path.

We conclude with a concise statement of the law of mixed-up-ness:

*Systems are partially ordered from states of less mixed-up-ness to states of greater mixed-up-ness by majorization. There are many paths from less mixed-up-ness to maximum mixed-up-ness. States in each allowed path are strictly ordered by mixed-up-ness rather than by entropy.*

## Data Availability

Not applicable.

## Abbreviations

The following abbreviations are used in this manuscript:

LOM	Law of Mixed-up-ness
SL	Second Law of Thermodynamics
YDL	Young Diagram Lattice
IP	Integer Partition
